# Function of the c-Met receptor tyrosine kinase in carcinogenesis and associated therapeutic opportunities

**DOI:** 10.1186/s12943-018-0796-y

**Published:** 2018-02-19

**Authors:** Yazhuo Zhang, Mengfang Xia, Ke Jin, Shufei Wang, Hang Wei, Chunmei Fan, Yingfen Wu, Xiaoling Li, Xiayu Li, Guiyuan Li, Zhaoyang Zeng, Wei Xiong

**Affiliations:** 1 The Key Laboratory of Carcinogenesis of the Chinese Ministry of Health, Xiangya Hospital, Central South University, Changsha, Hunan China; 2 The Key Laboratory of Carcinogenesis and Cancer Invasion of the Chinese Ministry of Education, Cancer Research Institute, Central South University, Changsha, Hunan China; 3 Hunan Key Laboratory of Nonresolving Inflammation and Cancer, Disease Genome Research Center, The Third Xiangya Hospital, Central South University, Changsha, Hunan China

**Keywords:** HGF/c-Met, PI3K/AKT, Ras/MAPK, Wnt, RON, EGFR, Therapeutic strategy

## Abstract

c-Met is a receptor tyrosine kinase belonging to the MET (MNNG HOS transforming gene) family, and is expressed on the surfaces of various cells. Hepatocyte growth factor (HGF) is the ligand for this receptor. The binding of HGF to c-Met initiates a series of intracellular signals that mediate embryogenesis and wound healing in normal cells. However, in cancer cells, aberrant HGF/c-Met axis activation, which is closely related to c-Met gene mutations, overexpression, and amplification, promotes tumor development and progression by stimulating the PI3K/AKT, Ras/MAPK, JAK/STAT, SRC, Wnt/β-catenin, and other signaling pathways. Thus, c-Met and its associated signaling pathways are clinically important therapeutic targets. In this review, we elaborate on the molecular structure of c-Met and HGF and the mechanism through which their interaction activates the PI3K/AKT, Ras/MAPK, and Wnt signaling pathways. We also summarize the connection between c-Met and RON and EGFR, which are also receptor tyrosine kinases. Finally, we introduce the current therapeutic drugs that target c-Met in primary tumors, and their use in clinical research.

## Background

c-Met (mesenchymal-epithelial transition factor), which belongs to the MET family, along with RON, is a type of receptor tyrosine kinase that is expressed on the surfaces of various epithelial cells; its ligand is HGF/SF(ligand hepatocyte growth factor/scatter factor) [1, 2]. HGF belongs to the soluble cytokine family and is also a member of the plasminogen-related growth factor family. It is synthesized by mesenchymal cells, fibroblasts, and smooth muscle cells, and acts through a paracrine mechanism to activate HGF/c-Met signaling to exert its biological functions [3]. Under normal conditions, HGF/c-Met can mediate embryogenesis, tissue regeneration, wound healing, and the formation of nerve and muscle, which is controlled by the tumor suppressor p53. Thus, this axis plays an important role in normal biological functions in humans [4–6].

However, as a type of proto-oncogene, abnormal activation of c-Met can promote the development and progression of multiple cancers such as liver, lung, colon, breast, pancreatic, ovarian, prostate, and gastric carcinomas, in addition to cancers of the nervous system such as glioblastoma [7–9]. The HGF/c-Met axis, which can interact and cooperate with other types of tyrosine kinases, can stimulate various downstream signaling pathways in tumor cells, such as PI3K/AKT, JAK/STAT, Ras/MAPK, SRC, and Wnt/β-catenin, among others [10–13]. These aforementioned phenomena regulate multiple biological processes such as tumor proliferation, invasion, metastasis, anti-apoptosis, EMT, and angiogenesis [14–17]. It has been determined that c-Met gene mutations, overexpression, and amplification also occur in a variety of human tumor types, and these events are closely related to the aberrant activation of the HGF/c-Met signaling pathway [18, 19]. Meanwhile, high c-Met expression is closely associated with poor prognosis in cancer patients. Studies have shown that abnormal activation of c-Met is critical for resistance to targeted therapies such as tyrosine kinase inhibitors and drugs that act against associated signaling pathways. Therefore, as abnormal c-Met function can increase the difficulty associated with tumor treatment, understanding its role in cancer is extremely important [4, 20].

## Structures of c-met and HGF

**Fig. 1:**
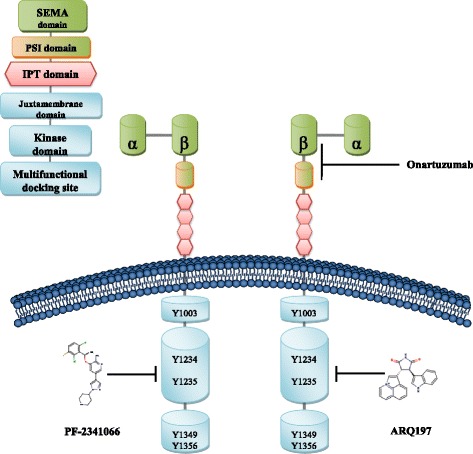
Structure of c-Met and binding sites for c-Met monoclonal antibody and small molecule inhibitors. c-Met is a heterodimer linked by an extracellular α chain and a transmembrane β chain. The β chain has a SEMA domain, a PSI domain, four IPT domains, a transmembrane domain, a juxtamembrane domain, a tyrosine kinase domain, and a C-terminal tail region. HGF is a heterodimer consisting of an α chain and a β chain linked via a disulfide bond, and forming six domains: the α chain contains a N-terminal hairpin domain and four Kringle domains and the β chain forms a serine protease analog domain lacking catalytic activity. The SEMA domain and the PSI domain in c-Met bind the β chain of HGF. The small molecule inhibitor PF-2341066 binds the TK domain of c-Met at Tyr312A, Lys345A, Pro317A, whereas the small molecule inhibitor ARQ197 forms a complex with the TK domain of c-Met at Pro1158A, Met1160A, Phe1123A, and onartuzumab forms a complex with the Sema-PSI domain of c-Met at Leu43B

The *MET* (c-Met encoding) gene is located on human chromosome 7 (7q21-q31), includes 21 exons and 20 introns, and encodes a protein that is approximately 120 kDa in size [21]. The translated product is processed to form a heterodimer that is linked by the extracellular α chain and the transmembrane β chain. The transmembrane chain consists of a SEMA domain (sema homology region; SEMA), a PSI domain (plexin-semaphorin-integrin; PSI), four IPT domains (immunoglobulin-like regions in plexins and transcription factors), a transmembrane domain, a juxtamembrane domain, a tyrosine kinase domain (TK domain), and a c-terminal docking site (carboxyl terminal; CT). SEMA is the site where HGF binds directly to c-Met, and PSI can stabilize this interaction. Ser-975 and Tyr-1003 sites at the juxtamembrane domain play an important role in the negative regulation of c-Met [14, 22, 23]. When HGF binds c-Met, Tyr-1234 and Tyr-1235 in the intracellular tyrosine kinase domain undergo autophosphorylation, which results in autophosphorylation of Tyr-1349 and Tyr-1356 in the C-terminal docking site. This facilitates the recruitment of intracellular effector molecules such as growth factor receptor-bound protein 2(GRB2), SRC, PI3K, and GAB1, and consequently the activation of downstream signaling pathways (Fig. 1) [24, 25].

The *HGF* gene encoding a 728-amino-acid protein is located on human chromosome 7 and consists of 18 exons and 17 introns [21]. Mature HGF is a heterodimer consisting of an α chain (69 kDa) and a β chain (34 kDa), which are linked by a disulfide bond. This protein consists of six domains. An N-terminal hairpin domain and four Kringle domains comprise the α chain, and the hairpin domain and first two Kringle domains are necessary for HGF to exert its biological function. The β chain forms a serine protease analog domain lacking catalytic activity, and this is the binding site for c-Met.

## HGF/c-met cascades in carcinoma

The binding of HGF to c-Met can initiate several downstream signaling pathways; we selected three significant pathways, based on their functions in carcinoma for futher review.

### HGF/c-met and the Ras pathway

**Fig. 2:**
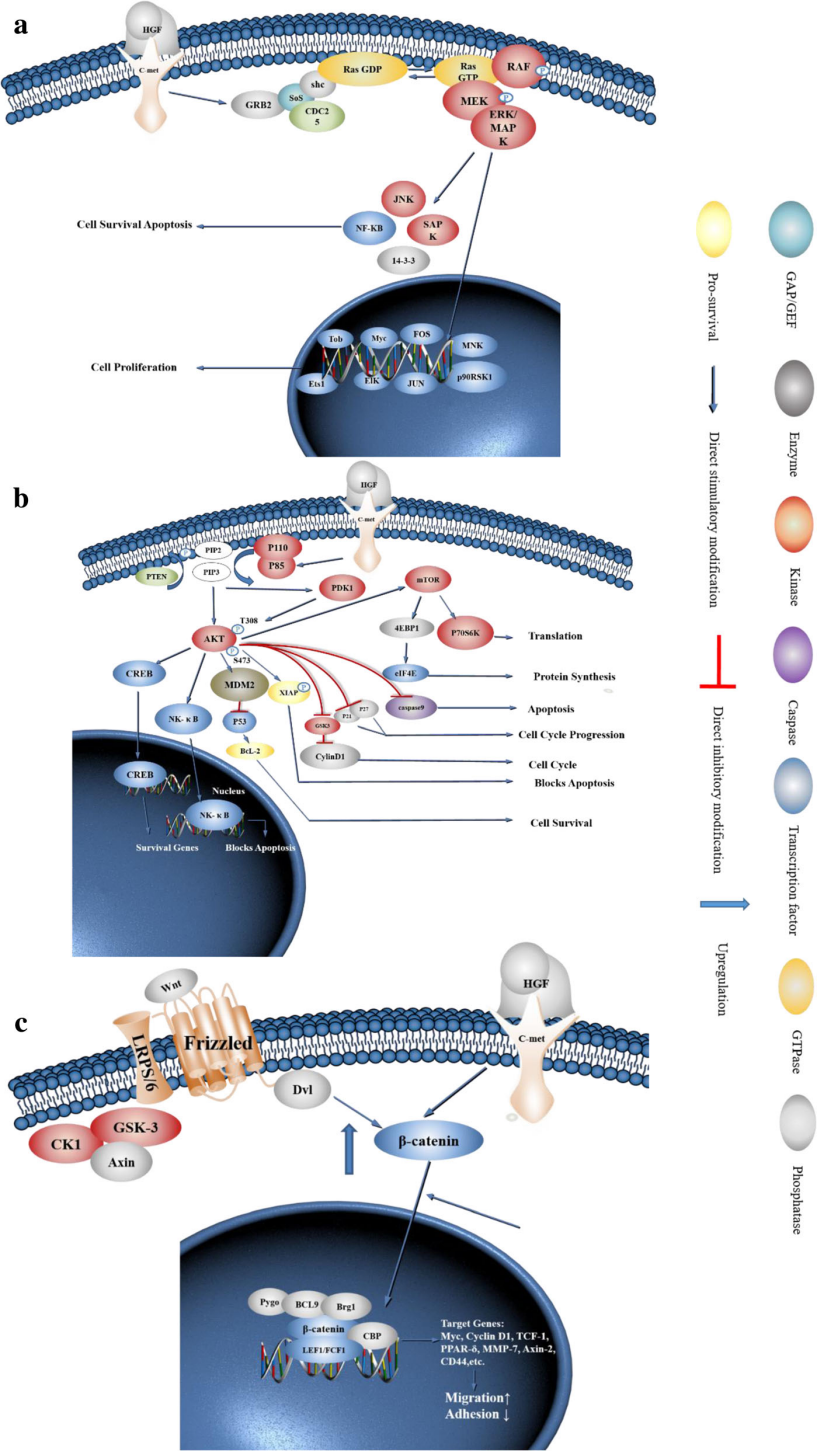
Illustration of the molecular mechanism of c-Met downstream signaling pathways. **a** Binding of HGF and c-Met can induce conformational changes in c-Met, resulting in the activation of downstream Ras-Raf-MAPK and PI3K/AKT/mTOR signaling pathways. After autophosphorylation, PTK binds Gab2 and activates it. Gab2 activates SOS; SOS activates Ras and then Ras stimulates Raf, MEK, and MAPKs. Activated MAPKs can enter the nucleus to regulate the expression of transcription factors such as Elk1, Etsl, and c-Myc (among others) to modulate cell proliferation and apoptosis. **b** The PTK domain is the site of autophosphorylation and also provides a docking site for PI3K. With this interaction, PI3K converts PIP2 to PIP3, and then PIP3 binds to the signaling proteins AKT and PDK1; PDK activates AKT, and activated AKT not only translocates to the nucleus, but also activates GSK-3 and mTOR to regulate the expression of multiple transcription factors. **c** Wnt binds to the low-density lipoprotein receptor-related protein 5/6/Frizzled (LRP5/LRP6/Frizzled) co-receptor group and activates the Dishevelled protein (DSH/Dvl) resulting in inhibition of the degradation of β-catenin by the destruction complex (consisting of Axin, adenomatosis polyposis coli (APC), protein phosphatase 2A (PP2A), glycogen synthase kinase 3 (GSK3) and casein kinase 1α (CK1α)).Subsequently, β-catenin is transported to the nucleus via Rac1 and other factors and binds to the LEF/TCF transcription factors in the nucleus with BCL9/LGS and Pygo to promote expression of oncogenes such as Myc, Cyclin D1, and MMP-7. This process can promote the invasion and migration of cancer cells. Aberrant activation of HGF/c-Met in tumor cells can block the degradation of β-catenin by the destruction complex, resulting in a higher concentration of β-catenin in the cytoplasm, and can also promote the entry of β-catenin into the nucleus

The binding of c-Met by its selective ligand HGF can induce structural changes in c-Met [26]; specifically, its intracellular protein tyrosine kinase (PTK) domain becomes activated, resulting in exposure of the multisubstrate docking site (MDS). Grb2 is then recruited to this site [27]. After autophosphorylation of the PTK domain, it can bind the SH2/SH3 domain of Grb2 [28], which subsequently recruits downstream guanine nucleotide exchange factors (GEFs) such as SOS. Downstream SOS can recruit Ras-GTP from the cell matrix to the membrane and convert it to activated Ras-GTP. Ras successively activates Raf, MEK, MAPKs, ERK, JNK (Jun N-terminal kinase), and p38 (HOG), among others, and the activated MAPKs then enter the cell nuclei to activate transcription factors (e.g. Elk1, Etsl, c-Myc) through phosphorylation. This, in turn, can interfere with the cell cycle and induce cell transformation, consequently promoting carcinogenesis. MAPKs also induce the degradation of proteins and matrix, promote cell migration, and sustain tumor proliferation (Fig. 2) [29, 30].

In tumor cells, the mutation rate of the *Ras* gene is approximately 25%, whereas in pancreatic cancer and colon cancer, the mutation rates could be 85 and 40%, respectively. Such mutations are predominantly point mutations and gene amplifications [27]. Mutations occur in codons 11, 12, 13,18, 59, and 69, which affect the interaction between Ras and GAP. Upon mutation, its intrinsic GTPase activity is inhibited, which can lead to malignant cell transformation through sustained activation of Ras2GTP (Fig. 2).

### HGF/c-met and PI3K pathway

When HGF binds c-Met and induces autophosphorylation, the phosphorylated residue acts as a docking site for the heterodimeric PI3K-p85 subunit. Here, the p85 subunit of phosphatidylinositol-3-kinase (PI3K) binds to the adaptor protein at the SH2/SH3 domain, using the same phosphorylated site. When PI3K recruits enough activated receptors, it initiates the phosphorylation of many phosphatidylinositol intermediates. Especially, in many tumor-associated signaling cascades, PI3K can convert phosphatidylinositol-4, 5-diphosphate (PIP2) to phosphatidylinositol-3,4,5-trisphosphate (PIP3). Phosphorylated RTKs can bind the SH2 domain of p85, and subsequently recruit the p85-p110 complex to cell membranes to activate the complex. Activated PI3K accelerates the conversion of PIP2 to PIP3. The association between PIP3 and signaling proteins containing a PH domain, namely, AKT and PDK1, facilitates the phosphorylation of AKT at Thr-308 and at Ser-473 by PDK1 [27]. Activated AKT, which later translocates to cell nuclei, modulates downstream transcription factors like FKHRL1, NF-κB, and Bcl-2, and inhibits the expression of tumor suppressor genes. AKT also phosphorylates GSK-3 and mammalian target of rapamycin (mTOR) or a series of inhibitory proteins such as p21CIP1 and p27KIP1; these, in turn, separately upregulate the expression of Cyclin D, shorten the cell cycle, and ultimately contribute to tumorigenesis [31]. In addition to this, RTKs might also activate the PI3K/AKT pathway through Ras (Fig. 2).

One study found that mTOR can regulate the degradation of extracellular matrix in cancer cells and influence the synthesis and secretion of matrix metalloproteinase; through this mechanism, this protein can also promote the invasion and metastasis of tumor cells [32]. Activated AKT might also phosphorylate nitric oxide synthase to produce NO, which positively regulates angiogenesis (Fig. 2).

The PI3K/AKT/mTOR pathway can modulate the expression of vascular endothelial growth factor (VEGF) and hypoxia inducible factor-1 (HIF-1) through the activation of human double minute 2 (HDM2) (Fig. 2) [33].

In addition, PTEN (phosphatase and tension homology deleted on chromosome 10) negatively regulates phosphorylation in the PI3K pathway. Specifically, this protein facilitates the dephosphorylation of PIP3, converting PIP3 to PIP2. Hence, it relieves the negative regulation of the downstream PI3K components AKT and mTOR. In tumor cells, mutations or deletions in PTEN are common, and enable the increased activation of the PI3K/AKT/mTOR pathway; this leads to aberrant activation of this pathway (Fig. 2).

### Association between the HGF/c-met and Wnt/β-catenin signaling pathways

HGF/c-Met is closely related to Wnt/β-catenin signaling, and promotes tumor proliferation, invasion, and metastasis by modulating this signaling pathway [34]. Studies have shown that in colon cancer and glioblastoma, c-Met expression can enhance Wnt/β-catenin signal transduction, and prevent GSK3β from phosphorylating β-catenin; this, in turn, promotes the translocation of β-catenin to the nucleus, facilitating tumorigenesis. Accordingly, it has been shown that c-Met inhibitors can inhibit Wnt pathway activity in tumor cells [35, 36]. Meanwhile, it has been found that in breast cancer cells undergoing osteolytic bone metastasis, the activation of HGF/c-Met signaling can promote β-catenin translocation to the nucleus and enhance its transcriptional activity. Therefore, HGF/c-Met can exert its biological function through the Wnt signaling pathway (Fig. 2) [37].

In normal cells lacking Wnt pathway activation, β-catenin is cytoplasmic and is phosphorylated at Ser-31, Ser-37, Thr-4, and Ser-45 by GSK3β and CK1 proteins, which are part of the destruction complex. At the same time, it can be acetylated by acetyltransferase p300/CBP-associated factor (PCAF) at Lys-49. Subsequently, these modified sites are recognized by and associate with the β-TrCP E3 ubiquitin ligase, resulting in its degradation by the proteasome, thereby preventing translocation to the nucleus [38, 39]. However, in tumor cells, aberrant activation of the HGF/c-Met pathway and stimulation of Wnt pathway block phosphorylation and acetylation of β-catenin through different signals. This results in the accumulation of β-catenin in the cytoplasm; it then enters the nucleus to displace Groucho, which has a transcriptional inhibitory effect on T-cell factor/lymphoid enhancer factor (TCF/LEF) transcription factors. β-catenin exerts its functions along with BCL9/LGS and Pygo to promote expression of Myc, Cyclin D1, and MMP-7, which facilitates proliferation, invasion, and metastasis (Fig. 2) [38, 40, 41].

## Crosstalk between c-met and other receptors tyrosine kinases

### C-met and RON

Studies have shown that c-Met and RON (receptor originated from nantes) are overexpressed [42] or aberrantly activated in many epithelial-derived malignant cancers [43–49]. These proteins can be involved in tumorigenesis by promoting cell proliferation, inhibiting apoptosis, enhancing angiogenesis, and promoting metastasis, among other functions, by acting upstream of these processes [46–49]. c-Met and RON can be activated by HGF and macrophage stimulating protein (MSP), respectively. Activated signaling depends on the tissue availability of adaptor proteins and signaling intermediates or the tendency of the adaptor proteins and signaling intermediates to undergo homodimerization or heterodimerization [50, 51]. MSP and HGF are highly homologous in sequence and structure [52], and are secreted as inactive single chains by multiple tissues and cells including smooth muscle, fibroblasts, adipose tissue, epithelial-derived tumors, liver, lungs, adrenal glands, placenta, and kidney. They are subsequently activated by proteasomal cleavage and form dimeric peptides consisting of α and β chains. In contrast to HGF, the high-affinity RON-binding site (for MSP) is located in the β chain [51].

The dimerization of these two monomers represents a major regulatory mechanism for the activation of tyrosine kinase receptors [53]. In some cases, the formation of a heterodimeric complex permits interaction and crosstalk between different receptors of the same subfamily. The epidermal growth factor receptor (EGFR) family is the best example of a tyrosine kinase receptor that undergoes homo and heterodimerization [54, 55]. Therefore, it is important to study the dimerization mechanism of PTKs. RON and c-Met are co-expressed in many types of tumors and crosstalk between c-Met and RON has been demonstrated [52]. Analysis of their structural homology suggested that they might interact, and in fact, studies have indicated that c-Met and RON can form heterodimers and phosphorylate each other [56]. One study showed that oncogenic addiction to c-Met requires co-expression of RON in four different tumor cell lines [50]. In these cases, RON was constitutively activated, and this was dependent on transphosphorylation by c-Met [50]. Experimentally, it has been shown that c-Met has stronger kinase activity than RON [57], and thus it is possible that heterodimers might be more efficiently activated than RON-RON homodimers. The fact that oncogenic addiction to c-Met requires RON implies that c-Met-RON heterodimers can promote the activation of diverse signaling cascades through different platforms. However, c-Met and RON possess remarkably similar tyrosine-binding sites that serve as docking sites for signaling molecules, and thus these signaling platforms might also be redundant. However, one study found that these two receptors have different kinase activities. Specifically, c-Met can be activated directly through Grb2 binding, but requires modulation for activation by other platforms [58]; in contrast, RON relies mainly on Grb2-associated binder (Gab1), based on the fact that the binding of Gab2 by RON attenuates the recruitment of Gab1 and represses signal transduction.

Grb2 has a unique role with respect to c-MET-RON heterodimers. Although Grb2 inhibits RON autophosphorylation, it enhances this process with c-MET [59]. Considering heterodimers of the EGFR family, the signaling diversity through heterodimers could depend on the relative abundance of each receptor [54].

RON expression might partially modulate c-Met activity, which can be applied when modeling this receptor. With respect to this, we found that knockdown of RON enhances the level and duration of HGF-mediated activation of MAPK and AKT [53]. Although the functional relevance of c-Met-RON heterodimers has not been fully explored, some studies suggest that general knockdown of RON leads to changes in c-Met signaling. For example, it was found that silencing RON in pancreatic cancer cell lines leads to upregulation of c-Met expression and activity [56]. This suggests that inhibitors that co-target or simultaneously block the kinase activities of both c-Met and RON might be clinically useful. However, most studies have not considered the possibility that separately inhibiting either c-Met or RON might lead to compensation by [60] the other.

### C-met and EGFR

It has been confirmed that signal transduction between the c-Met and EGFR pathways is closely linked in breast cancer, lung cancer, brain cancer, and other tumors; however, the associated mechanism is still not fully understood [61–64]. Studies have shown that 70% of EGFR-activating mutations in non-small cell lung carcinoma (NSCLC) are associated with an initial positive response to the EGFR inhibitors gefitinib or erlotinib [65]. However, the vast majority of tumors that respond to EGFR inhibitors achieve acquired resistance [66]. Interestingly, the expression and activation of c-Met are associated with initial resistance and acquired resistance to EGFR inhibitors in patients with NSCLC [66–68]. Initial resistance might occur through the simultaneous activation of c-Met and EGFR pathways in lung cancer, whereas inhibiting both maximizes the inhibitory effect on the tumor [61]. As such, studies have shown that c-Met might be an effective therapeutic target for overcoming EGFR inhibitor resistance in lung cancer [62].

Possible explanations regarding this mechanism are as follows. One study has already shown that the second mutation in EGFR, T790 M, and the amplification of the MET proto-oncogene will lead to the activation of its downstream ERBB3-initiated PI3K/AKT pathway, resulting in EGFR-TKI acquired resistance [67, 69, 70]. When the c-MET gene is amplified, the two downstream pathways (Grb2/MAPK and PI3K/AKT) are activated by the increase in the number of ERBB3 receptors [69, 70].

In addition, continuous interaction with HGF facilitates c-Met amplification-mediated reversible resistance to EGFR-TKI treatment [66, 70]. When HGF activates Met, it activates MAPK and PI3K/AKT signaling pathways through Gab1, leading to the occurrence of irreversible EGFR-TKI resistance [66].

**Fig. 3:**
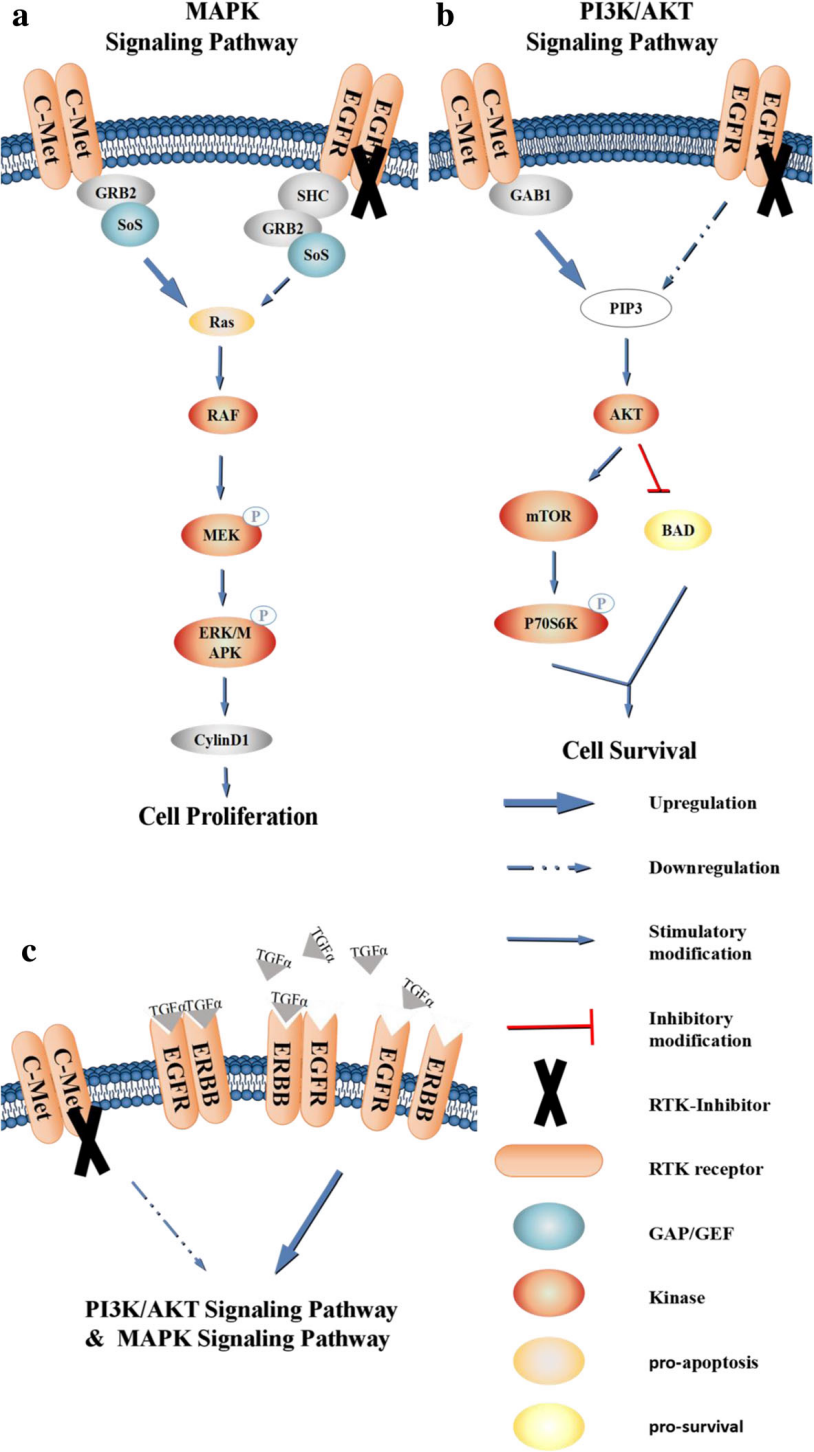
Crosstalk between c-Met and EGFR. **a, b** The tyrosine kinase receptors EGFR and c-Met can initiate downstream PI3K/Akt signaling resulting in anti-apoptotic processes and Grb2/MAPK activation to promote the proliferation of tumor cells. Therefore, it is speculated that there might be an effect that allows c-Met to bypass the EGFR receptor to activate its downstream pathway, resulting in resistance to EGFR-TKI monotherapy. **c** c-Met-TKI monotherapy triggers upregulation of the EGFR ligand TGF-α, as well as upregulation of the EGFR protein family receptor ErbB3, which can contribute to one of the most potent dimers that can activate c-Met downstream pathways leading to acquired resistance in cancer cells

If EGFR and Met mutations exist simultaneously, drug resistance will be further exacerbated [70]. Therefore, we speculate that c-Met activation of downstream PI3K/AKT and MAPK pathways bypasses EGFR activation because they can both act as tyrosine kinase receptors and activate this pathway (Fig. 3). In addition, c-Met can either directly or indirectly transactivate the PI3K pathway; the fact that c-Met is not activated by this RTK also supports this hypothesis [71].

Another study found that EGFR mutation and Met activation were observed in tumor cells. At the same time, whereas the activation of c-Met was not the result of gene mutation, it resulted in poor prognosis for NSCLC metastasis [68]. In addition, after reversible resistance to EGFR-TKIs in lung cancer cells, HGF can induce an irreversible second mutation (Fig. 3) [66].

HGF/c-Met is activated in approximately 50% of hepatocellular carcinomas (HCC), and expression levels of these proteins are associated with poor clinical prognosis for this disease [72–75]. Cells with constitutive c-Met activity respond to c-Met inhibition [76]; however, one study found that monotherapy does not completely eliminate tumor growth, suggesting that tumor survival mechanisms that bypass the inhibition of this pathway might be involved in the maintenance of tumor growth in response to these treatments [77].

In previous studies, inhibition of the EGFR pathway was shown to lead to either activation or inhibition of the c-Met pathway, whereas another study showed that c-Met inhibition leads to the activation of the EGFR pathway in a c-Met-positive HCC model [76]. In addition, EGFR inhibitor monotherapies are not significantly effective with respect to in vitro cell viability [76]. c-Met inhibitor monotherapy triggers several survival mechanisms that bypass cell death induced by these agents, including increased expression of the EGFR ligand TGF-α and ErbB3. It has been determined that members of the EGFR family can form homodimers or heterodimers and that different dimers have different signal transduction capabilities; specifically, ErbB3 can heterodimerize with ErbB1 to form one of the most potent dimers [78]. Experiments have shown that c-Met inhibition enhances EGFR signaling by increasing ErbB3 expression [76]. In addition, the increase in TGF-α expression that results from c-Met inhibition, whether this occurs through an autocrine or paracrine mechanism, and its effect on HCC cell survival requires further study.

## Current clinical trials targeting c-met

Currently, drugs targeting c-Met that are in clinical trials can be classified as monoclonal antibodies (e.g. onartuzumab) and small molecule inhibitors. Small molecule inhibitors bind to the tyrosine kinase domain of c-Met and can be further classified into ATP competitive inhibitors (e.g. crizotinib) and non-ATP competitive inhibitors (e.g. tivantinib). Inhibitors belonging to the same group inhibit c-Met downstream signaling in a similar manner. Therefore, in this review, we will introduce current clinical trials targeting c-Met, and have chosen onartuzumab, crizotinib, and tivantinib as examples to elaborate on their c-Met binding sites, as shown in Fig. 1.

### Anti-c-met monoclonal antibody Metmab (onartuzumab)

Onartuzumab is a humanized single-armed specific monoclonal antibody targeting c-Met. The binding of onartuzumab to c-Met is highly specific and this antibody can block c-Met-HGF binding specifically by blocking the HGF α-chain and by forming a complex with the Sema-PSI domain of c-Met [79]; this process occurs without exerting an agonistic activity or triggering c-Met dimerization.

Onartuzumab has been applied as a c-Met inhibitor for the treatment of NSCLC and breast cancer in clinical trials (Table 1) [80], and it proved to be considerably effective. Other studies also found that onartuzumab in combination with erlotinib and placebo is effective for NSCLC. Therefore, this drug might have potential to treat c-Met-overexpressing cancer.

**Table 1:** Ongoing studies with Metmb (onartuzumab)

NCT Number	Combination Drugs	PHASES	URL
NCT01897038	Drug: Onartuzumab	Phase1	https://ClinicalTrials.gov/show/NCT01897038
	Drug: Sorafenib		
NCT01519804	Drug: Placebo	Phase2	https://ClinicalTrials.gov/show/NCT01519804
	Drug: cisplatin/carboplatin		
	Drug: onartuzumab		
	Drug: paclitaxel		
NCT00854308	Drug: Erlotinib HCl	Phase2	https://ClinicalTrials.gov/show/NCT00854308
	Drug: MetMAb		
	Drug: placebo (0.9% saline)		
NCT01496742	Drug: Placebo	Phase2	https://ClinicalTrials.gov/show/NCT01496742
	Drug: RO5490258		
	Drug: bevacizumab [Avastin]		
	Drug: cisplatin/carboplatin		
	Drug: paclitaxel		
	Drug: pemetrexed		
NCT02031744	Drug: erlotinib [Tarceva]	Phase3	https://ClinicalTrials.gov/show/NCT02031744
	Drug: Placebo		
	Drug: Onartuzumab [MetMAb]		
NCT01887886	Drug: erlotinib	Phase3	https://ClinicalTrials.gov/show/NCT01887886
	Drug: onartuzumab		
	Drug: placebo		
NCT01456325	Drug: Erlotinib	Phase 3	https://ClinicalTrials.gov/show/NCT01456325
	Drug: Onartuzumab (MetMab)		
	Drug: Placebo		

### Small molecule inhibitors

#### Crizotinib

Crizotinib (PF-02341066, trade name: Xalkori), an effective small molecule inhibitor of c-Met, was derived from the first-generation series c-Met inhibitor, PHA-66752 (3-benzyloxy-2-amino). PF-22341066 targets the TK domain of c-Met, and after a series of reactions, some residues cause a conformational change, which interferes with the ATP binding site. One clinical trial (phase I) for the treatment of NSCLC with enhanced Met amplification, performed in 2014 [81], showed that crizotinib has increased potential for the treatment of c-Met-associated cancer. Crizotinib is one of five drugs approved by the FDA for the treatment of advanced NSCLC, to date [82], and it is used for the clinical treatment of ROS1-positive lung cancer [83]. Moreover, the number of studies focusing on the combination of crizotinib and other drugs is increasing. Huang et al. [84] showed that crizotinib with cisplatin induces G2/M cell cycle arrest and apoptosis in ovarian cancer cells. Stanley et al. [85] elaborated on the different growth inhibitory effects caused by the combination of c-Met inhibitors with cytotoxic drugs using breast cancer cell lines (BT474, MCF7, MDA-MB-468, and SKBr3). Results suggested that crizotinib and EGFR-TKIs might have a synergistic effect on MCF7 and MDA-MB-468 cells and an antagonistic effect on BT474 and SKBr3 cells. The combination of EGFR-TKIs and crizotinib was shown to have a more pronounced effect than a single drug regimen on breast cancer. In addition, sensitivity to mitomycin C (MMC), when combined with crizotinib, was studied using a colorectal cancer cell line. The results also showed that a combination of the two drugs resulted in increased tumor cell apoptosis and a synergistic effect. Currently, several clinical trials are in progress. A summary of these trials is provided in Table 2.

**Table 2:** Ongoing studies with crizotinib

NCT Number	Combination Drugs	PHASES	URL
NCT00932451	Drug: PF-02341066	Phase2	https://ClinicalTrials.gov/show/NCT00932451
NCT00932893	Drug: PF-02341066	Phase3	https://ClinicalTrials.gov/show/NCT00932893F
	Drug: Pemetrexed		
	Drug: Docetaxel		
NCT01154140	Drug: treatment	Phase3	https://ClinicalTrials.gov/show/NCT01154140
	Drug: treatment		
NCT01685060	Drug: LDK378	Phase2	https://ClinicalTrials.gov/show/NCT01685060
NCT01121575	Drug: PF-02341066	Phase1	https://ClinicalTrials.gov/show/NCT01121575
	Drug: PF-00299804		
	Drug: PF-02341066		
	Drug: PF-00299804		
NCT00965731	Drug: Erlotinib	Phase1	https://ClinicalTrials.gov/show/NCT00965731
	Drug: PF-02341066		
NCT02435108	Drug: crizotinib	Phase2	https://ClinicalTrials.gov/show/NCT02435108

#### Cabozantinib

Cabozantinib (XL184) is a small molecule inhibitor of Met and AXL [86, 87], and has been approved by the FDA for the treatment in progressive metastatic thyroid medullary carcinoma [60, 88], and also for advanced renal cell carcinoma after the implementation of antiangiogenic therapy regimens [89]. Wakelee divided NSCLC patients into three groups as follows: the first two groups were administered erlotinib alone (150 mg poqd) and cabozantinib alone (60 mg poqd), whereas the third group was administered combination therapy (150 mg erlotinib/40 mg cabozantinib). Results showed that progression-free survival and overall survival were significantly improved with cabozantinib treatment. Shotani et al. [90] showed that cabozantinib is effective in inhibiting growth and invasion in BCa cell lines driven by HGF (5637 and T24), and blocked HGF-Met signaling to inhibit MMP1 expression. Thus, cabozantinib has potential for the treatment of muscle invasive bladder cancer (MIBC). At present, this drug is at the clinical stage for prostate cancer treatment, and has been tested in phase II trials.

#### Foretinib

Foretinib (GSK1363089) is an ATP-competitive c-Met inhibitor, and its therapeutic potential has been assessed for different tumors including head and neck cancer, gastric cancer, and liver cancer [56, 57, 91, 92]. Chia et al. [93] conducted a phase I study to determine the effect of combining foretinib with lapatinib on HER-2-positive metastatic breast cancer. The study suggested that the combined use of foretinib and lapatinib at doses of 45 mg and 1000 mg PO, respectively, could be tolerated relatively well. The most common grade 3 and higher toxic adverse reactions were mainly high blood pressure, diarrhea, nausea, and fatigue. Yin et al. [22] also demonstrated that foretinib inhibits prostate cancer (PCa) metastasis by targeting c-Met.

#### LY280163

LY280163 is an ATP competitive Met tyrosine kinase inhibitor developed by Lilly. Cheng et al. [94] showed that this drug can improve the response of MEK inhibitors such as trametinib in metastatic uveal melanoma (UM) patients and promote the expression of PARP. In addition, studies [95] have investigated the effect of LY2801653 on human cholangiocarcinoma (CCC) cell lines. Using a xenograft mouse model, it was determined that LY2801653 blocks c-Met phosphorylation, down-regulates downstream target expression, and inhibits CCC cell proliferation and xenograft tumor growth.

#### MK2461

MK2461 is an ATP competitive small molecule multi-target inhibitor developed by Merck Sharp & Dohme Corp. It is effective in inhibiting the proto-oncogene c-Met mutants N1100Y, Y1230C, Y1230H, Y1235D, and M1250Tn1100y. Currently, this drug is in experimental stage I clinical trials for advanced cancer.

#### Capmatinib

Capmatinib (INC280) blocks c-Met phosphorylation and the activation of key downstream molecules in c-Met-dependent tumor cell lines, causing mitochondrial membrane depolarization and DNA repair [96, 97]. The drug has been utilized in phase I trials for advanced solid cancer. Wei et al. [96] found that the addition of capmatinib could effectively block cell proliferation induced by cancer associated fibroblast (CAF) matrix with overexpression of HGF, and could eliminate CAF-induced ovarian cancer cell resistance. The latest study by Lara et al. [97] utilized a series of NSCLC cell lines (including three EGFR-mutant cell lines, HCC827, PC9 and H1975, one Kirsten rat sarcoma virus oncogene mutant cell line, H358, and one EGFR and KRAS wild type cell line, H1666) to determine whether capmatinib in combination with erlotinib could attenuate erlotinib resistance. The Massachusetts General Hospital in the United States has also launched a clinical trial for the use of capmatinib in stage IV patients with malignant NSCLC. In addition, Novartis Pharmaceuticals is performing clinical phase II trials using oral capmatinib combined with gefitinib for NSCLC patients with c-Met amplification.

#### Tivantinib

Tivantinib (ARQ197), developed by American ArQule Corporation and Japan’s Daiichi Sankyo and Kyowa Hakko Kogyo, is a non-ATP competitive inhibitor that blocks receptor activation and downstream signaling by binding to unactivated receptors [49, 98]. ARQ197 directly binds the A-loop and P-loop phenylalanines by inducing “hydrophobic collapse”, resulting in disruption of the ionic interaction in the catalytic residue with the help of Arg1227, Tyr1230, and other residues. In recent years, its pharmacokinetic mechanism has become increasingly controversial. It was previously believed that tivantinib can exert its biological effects by directly inhibiting c-Met receptor tyrosine kinases. However, several subsequent studies showed that the biological effect of tivantinib does not depend on the c-met receptor; in contrast, it inhibits tumor cells through microtubule depolymerization. Tivantinib inhibits tubulin polymerization, disrupting tubulin metabolism, prolonging cell G2/M phase, and promoting apoptosis [99, 100]. Studies have shown that combining tivantinib with erlotinib for treatment improves progression free survival (PFS) and is well tolerated [101–103]. In patients with advanced solid tumors, tivantinib combined with sorafenib treatment was shown to be safe, especially for renal cell carcinoma (RCC), hepatocellular carcinoma (HCC), and melanoma patients harboring tumors with high levels of c-Met; an enhanced therapeutic effect was also observed for these cases. The combination of the two drugs was shown to enhance the antitumor activity of sorafenib, thus reducing associated resistance without promoting off-target effects [104]. As is described in Table 3, we summarized ongoing studies involving tivantinib.

**Table 3:** Ongoing studies with tivantinib

NCT Number	Combination Drugs	PHASES	URL
NCT01755767	Drug:Tivantinib	Phase 3	https://ClinicalTrials.gov/show/NCT01755767
	Drug: Placebo		
NCT00988741	Drug: ARQ 197	Phase 2	https://ClinicalTrials.gov/show/NCT00988741
	Drug: Placebo		
NCT01656265	Drug: ARQ 197	Phase 1	https://ClinicalTrials.gov/show/NCT01656265
NCT00802555	Drug: ARQ 197	Phase 1	https://ClinicalTrials.gov/show/NCT00802555
NCT00557609	Drug: ARQ 197	Phase 2	https://ClinicalTrials.gov/show/NCT00557609
NCT01575522	Other: Laboratory Biomarker Analysis	Phase 2	https://ClinicalTrials.gov/show/NCT01575522
	Drug: Tivantinib		
NCT01395758	Drug: ARQ 197 plus erlotinib	Phase 2	https://ClinicalTrials.gov/show/NCT01395758
	Drug: Pemetrexed, docetaxel or gemcitabine		
NCT01244191	Drug: Tivantinib	Phase 3	https://ClinicalTrials.gov/show/NCT01244191
	Drug: Placebo		
	Drug: Erlotinib		
NCT01069757	Drug: ARQ 197 and Erlotinib	Phase 1	https://ClinicalTrials.gov/show/NCT01069757
NCT01251796	Drug: ARQ 197 and Erlotinib	Phase 1	https://ClinicalTrials.gov/show/NCT01251796
NCT00777309	Drug: ARQ 197	Phase 2	https://ClinicalTrials.gov/show/NCT00777309
	Drug: Erlotinib		
	Drug: Placebo		

## Conclusions

Despite research on c-Met over the past 30 years, the structure and function of this tyrosine kinase has not been well established. HGF/c-Met mediates cascades that play a key role in tumorigenesis; extensive research on those pathways is not only beneficial for enhancing our understanding of the mechanisms associated with carcinoma, but also suggest promising targets for the development of novel cancer treatments. Considering the complexity of the HGF/c-Met axis, further exploration of the mechanism through which blocking c-Met activation modulates downstream pathways is required.

Recently, many clinical trials have found that drug resistance is more easily acquired with single drug therapy; therefore, research on combining c-Met inhibitors with other drugs (e.g. EGFR-TKI) will lead to the rapid discovery of effective treatment options. One study showed that in a nude mouse model of treatment-sensitive NSCLC, erlotinib resistance could be effectively reversed by the administration of SU1274. Meanwhile, Klempner et al. [105] found that cabozantinib could reverse resistance to crizotinib. Thus, the use of combinations of drugs to avoid resistance induced by the utilization of a single drug might become a major priority for researchers developing novel c-Met inhibitors.

Recently, phase II/III clinical trials for c-Met inhibitors have been initiated, and many of these drugs are regarded as second-line drugs. The main problem is that in most c-Met-overexpressing cancer cells, this receptor is not always the only driver of carcinoma, as it often interacts with other tyrosine kinase receptors. For example, the cytotoxic effect of tivantinib is not due solely to c-Met inhibition. Meanwhile, non-specific reactions are also a major problem when using c-Met inhibitors. For example, the c-Met monoclonal antibody Metmab is associated with several adverse events including rash, diarrhea, fatigue, and nausea/vomiting.

Under these circumstances, more precise information regarding how the drug functions and its relationship with c-Met and other tyrosine kinase receptors is required. In fact, as mentioned previously, the relationship between c-Met and its family member RON is being extensively studied, whereas the mechanism underlying the crosstalk between c-Met and RON is still not fully understood. One study showed that in pancreatic cancer, silencing RON might modulate the c-Met signaling pathway, resulting in a compensatory reaction during the downregulation of either tyrosine kinase receptor [56–59]. As such, we might consider targeting c-Met and RON simultaneously. It turns out that c-Met and RON also interact with other tyrosine kinase receptors. Nevertheless, compared to RON, these other receptors might not be as indispensable for the activation of c-Met, and the significance of this crosstalk is still not well understood.

Meanwhile, the initiation of carcinoma can be induced by multiple factors including genome backgrounds, environmental factors [106], microenvironment [29, 107], even the non-coding RNAs [108–110]. Considering that, further efforts on the interactions between c-Met and other cancer related risk factors will be necessary in promoting the process of precise medical treatment on c-Met which thus, demand a further comprehensive understanding of this tyrosine kinase receptor.

However, based on the fact that c-Met has an intimate association with cancer, targeting this receptor for the treatment of tumorigenesis is still thought to be associated with vast clinical significance.

## Abbreviations

HCC: Hepatocellular carcinoma
APC: Adenomatosis polyposis coli
CAF: Cancer associated fibroblast
CCC: Cholangiocarcinoma
CK1α: Casein kinase 1α
c-Met: Mesenchymal-epithelial transition factor
DSH/Dvl: Dishevelled protein
EGFR: Epidermal growth factor receptor
ErbB: Erb-b2 receptor tyrosine kinase 4
Gab1: Grb2-associated binder
GEFs: Guanine nucleotide exchange factors
GRB2: Growth factor receptor-bound protein 2
GSK3: Glycogen synthase kinase 3
HCC: Hepatocellular carcinoma
HDM2: Human double minute 2
HGF/SF: Hepatocyte growth factor/scatter factor
HIF-1: Hypoxia inducible factor-1
IPT domains: Immunoglobulin-like regions in plexins and transcription factors
JNK: Jun N-terminal kinase
LRP5/LRP6/Frizzled: Low-density lipoprotein receptor-related protein 5/6/Frizzled
MDS: Multisubstrate docking site
MET: MNNG HOS transforming gene
MIBC: Muscle invasive bladder cancer
MMC: Mitomycin C
MSP: Macrophage stimulating protein
MTOR: Mammalian target of rapamycin
NSCLC: Non-small cell lung carcinoma
PCa: Prostate cancer
PCAF: Acetyltransferase p300/CBP-associated factor
PFS: Progression free survival
PHA-66752: 3-benzyloxy-2-amino
PI3K: Phosphatidylinositol-3-kinase
PIP2: Phosphatidylinositol-4, 5-diphosphate
PIP3: Phosphatidylinositol-3,4,5-triphosphate
PP2A: Protein phosphatase 2A
PSI domain: Plexin-semaphorin-integrin
PTEN: Phosphatase and tension homology deleted on chromosome 10
PTK: Protein tyrosine kinase
RCC: Renal cell carcinoma
RON: Receptor originated from nantes
SEMA domain: Sema homology region
TCF/LEF: T-cell factor/lymphoid enhancer factor
UM: Uveal melanoma
VEGF: Vascular endothelial growth factor

## References

[1] Salgia R (2017). MET in lung cancer: biomarker selection based on scientific rationale. Mol Cancer Ther.

[2] Fu YT, Zheng HB, Zhou L, Zhang DQ, Liu XL, Sun H (2017). Valproic acid, targets papillary thyroid cancer through inhibition of c-Met signalling pathway. Am J Transl Res.

[3] Rucki AA, Xiao Q, Muth S, Chen J, Che X, Kleponis J, Sharma R, Anders RA, Jaffee EM, Zheng L. Dual Inhibition of Hedgehog and c-Met Pathways for Pancreatic Cancer Treatment. Mol Cancer Ther. 2017;16:2399-409. 10.1158/1535-7163.MCT-16-0452 PMC5670001 28864680

[4] Zhu L, Xiong X, Kim Y, Okada N, Lu F, Zhang H, Sun H (2016). Acid sphingomyelinase is required for cell surface presentation of met receptor tyrosine kinase in cancer cells. J Cell Sci.

[5] Liu WT, Jing YY, Yu GF, Chen H, Han ZP, Yu DD, Fan QM, Ye F, Li R, Gao L (2016). Hepatic stellate cell promoted hepatoma cell invasion via the HGF/c-Met signaling pathway regulated by p53. Cell Cycle.

[6] Zhang Y, Du Z, Zhang M (2016). Biomarker development in MET-targeted therapy. Oncotarget.

[7] Szturz P, Raymond E, Abitbol C, Albert S, de Gramont A, Faivre S (2017). Understanding c-MET signalling in squamous cell carcinoma of the head & neck. Crit Rev Oncol Hematol.

[8] Kim B, Jung N, Lee S, Sohng JK, Jung HJ (2016). Apigenin inhibits cancer stem cell-like phenotypes in human Glioblastoma cells via suppression of c-Met Signaling. Phytother Res.

[9] International Cancer Genome Consortium PedBrain Tumor P (2016). Recurrent MET fusion genes represent a drug target in pediatric glioblastoma. Nat Med.

[10] Imura Y, Nakai T, Yamada S, Outani H, Takenaka S, Hamada K, Araki N, Itoh K, Yoshikawa H, Naka N (2016). Functional and therapeutic relevance of hepatocyte growth factor/c-MET signaling in synovial sarcoma. Cancer Sci.

[11] Liang Y, Liu J, Liu T, Yang X (2017). Anti-c-Met antibody bioconjugated with hollow gold nanospheres as a novel nanomaterial for targeted radiation ablation of human cervical cancer cell. Oncol Lett.

[12] Pilotto S, Carbognin L, Karachaliou N, Ma PC, Rosell R, Tortora G, Bria E (2017). Tracking MET de-addiction in lung cancer: a road towards the oncogenic target. Cancer Treat Rev.

[13] Wu JC, Wang CT, Hung HC, Wu WJ, Wu DC, Chang MC, Sung PJ, Chou YW, Wen ZH, Tai MH (2016). Heteronemin is a novel c-Met /STAT3 inhibitor against advanced prostate cancer cells. Prostate.

[14] Safaie Qamsari E, Safaei Ghaderi S, Zarei B, Dorostkar R, Bagheri S, Jadidi-Niaragh F, Somi MH, Yousefi M (2017). The c-Met receptor: implication for targeted therapies in colorectal cancer. Tumour Biol.

[15] Hughes PE, Rex K, Caenepeel S, Yang Y, Zhang Y, Broome MA, Kha HT, Burgess TL, Amore B, Kaplan-Lefko PJ (2016). In vitro and in vivo activity of AMG 337, a potent and selective MET Kinase inhibitor, in MET-dependent cancer models. Mol Cancer Ther.

[16] Kuang W, Deng Q, Deng C, Li W, Shu S, Zhou M (2017). Hepatocyte growth factor induces breast cancer cell invasion via the PI3K/Akt and p38 MAPK signaling pathways to up-regulate the expression of COX2. Am J Transl Res.

[17] Leung E, Xue A, Wang Y, Rougerie P, Sharma VP, Eddy R, Cox D, Condeelis J (2017). Blood vessel endothelium-directed tumor cell streaming in breast tumors requires the HGF/C-Met signaling pathway. Oncogene.

[18] Bahrami A, Shahidsales S, Khazaei M, Ghayour-Mobarhan M, Maftouh M, Hassanian SM, Avan A (2017). c-Met as a potential target for the treatment of gastrointestinal cancer: current status and future perspectives. J Cell Physiol.

[19] Barrow-McGee R, Kishi N, Joffre C, Menard L, Hervieu A, Bakhouche BA, Noval AJ, Mai A, Guzman C, Robert-Masson L (2016). Beta 1-integrin-c-Met cooperation reveals an inside-in survival signalling on autophagy-related endomembranes. Nat Commun.

[20] Caenepeel S, Cooke K, Wadsworth S, Huang G, Robert L, Moreno BH, Parisi G, Cajulis E, Kendall R, Beltran P (2017). MAPK pathway inhibition induces MET and GAB1 levels, priming BRAF mutant melanoma for rescue by hepatocyte growth factor. Oncotarget.

[21] Li Y, Liu H, Chen J (2014). Dysregulation of HGF/ c-Met signal pathway and their targeting drugs in lung cancer. Zhongguo Fei Ai Za Zhi.

[22] Yin B, Liu Z, Wang Y, Wang X, Liu W, Yu P, Duan X, Liu C, Chen Y, Zhang Y (2017). RON and c-Met facilitate metastasis through the ERK signaling pathway in prostate cancer cells. Oncol Rep.

[23] Hass R, Jennek S, Yang Y, Friedrich K (2017). c-Met expression and activity in urogenital cancers - novel aspects of signal transduction and medical implications. Cell Commun Signal.

[24] Al-U'datt DGF, Al-Husein BAA, Qasaimeh GR (2017). A mini-review of c-Met as a potential therapeutic target in melanoma. Biomed Pharmacother.

[25] Cecchi F, Rabe DC, Bottaro DP (2010). Targeting the HGF/met signalling pathway in cancer. Eur J Cancer.

[26] Cecchi F, Rabe DC, Bottaro DP (2012). Targeting the HGF/met signaling pathway in cancer therapy. Expert Opin Ther Targets.

[27] Rosário M (2003). How to make tubes: signaling by the met receptor tyrosine kinase. Trends Cell Biol.

[28] Vogel W, Ullrich A (1996). Multiple in vivo phosphorylated tyrosine phosphatase SHP-2 engages binding to Grb2 via tyrosine 584. Cell Growth Differ.

[29] Wang M, Zhao J, Zhang L, Wei F, Lian Y, Wu Y, Gong Z, Zhang S, Zhou J, Cao K (2017). Role of tumor microenvironment in tumorigenesis. J Cancer.

[30] Liebmann C (2001). Regulation of MAP kinase activity by peptide receptor signalling pathway: paradigms of multiplicity. Cell Signal.

[31] Shaw RJ, Cantley LC (2006). Ras, PI(3)K and mTOR signalling controls tumour cell growth. Nature.

[32] Mottet D, Dumont V, Deccache Y, Demazy C, Ninane N, Raes M, Michiels C (2003). Regulation of hypoxia-inducible factor-1alpha protein level during hypoxic conditions by the phosphatidylinositol 3-kinase/Akt/glycogen synthase kinase 3beta pathway in HepG2 cells. J Biol Chem.

[33] Jiang BH, Liu LZ (2008). AKT signaling in regulating angiogenesis. Curr Cancer Drug Targets.

[34] Holland JD, Gyorffy B, Vogel R, Eckert K, Valenti G, Fang L, Lohneis P, Elezkurtaj S, Ziebold U, Birchmeier W (2013). Combined Wnt/beta-catenin, met, and CXCL12/CXCR4 signals characterize basal breast cancer and predict disease outcome. Cell Rep.

[35] Tuynman JB, Vermeulen L, Boon EM, Kemper K, Zwinderman AH, Peppelenbosch MP, Richel DJ (2008). Cyclooxygenase-2 inhibition inhibits c-Met kinase activity and Wnt activity in colon cancer. Cancer Res.

[36] Kim KH, Seol HJ, Kim EH, Rheey J, Jin HJ, Lee Y, Joo KM, Lee J, Nam DH (2013). Wnt/beta-catenin signaling is a key downstream mediator of MET signaling in glioblastoma stem cells. Neuro-Oncology.

[37] Previdi S, Maroni P, Matteucci E, Broggini M, Bendinelli P, Desiderio MA (2010). Interaction between human-breast cancer metastasis and bone microenvironment through activated hepatocyte growth factor/met and beta-catenin/Wnt pathways. Eur J Cancer.

[38] Tortelote GG, Reis RR, de Almeida MF, Abreu JG (2017). Complexity of the Wnt/betacatenin pathway: searching for an activation model. Cell Signal.

[39] Wu C, Zhuang Y, Jiang S, Liu S, Zhou J, Wu J, Teng Y, Xia B, Wang R, Zou X (2016). Interaction between Wnt/beta-catenin pathway and microRNAs regulates epithelial-mesenchymal transition in gastric cancer (review). Int J Oncol.

[40] Yan Q, Zeng Z, Gong Z, Zhang W, Li X, He B, Song Y, Li Q, Zeng Y, Liao Q (2015). EBV-miR-BART10-3p facilitates epithelial-mesenchymal transition and promotes metastasis of nasopharyngeal carcinoma by targeting BTRC. Oncotarget.

[41] Arend RC, Londono-Joshi AI, Straughn JM, Buchsbaum DJ (2013). The Wnt/beta-catenin pathway in ovarian cancer: a review. Gynecol Oncol.

[42] Mariani M, McHugh M, Petrillo M, Sieber S, He S, Andreoli M, Wu Z, Fiedler P, Scambia G, Shahabi S, Ferlini C (2014). HGF/c-Met axis drives cancer aggressiveness in the neo-adjuvant setting of ovarian cancer. Oncotarget.

[43] Raman JD (2008). Re: prognostic value of MET, RON and histoprognostic factors for urothelial carcinoma in the upper urinary tract. E. Comperat, M. Roupret, E. Chartier-Kastler, M. O. Bitker, F. Richard, P. Camparo, F. Capron and O. Cussenot. J Urol 2008; 179: 868-872. J Urol.

[44] Cheng HL, Liu HS, Lin YJ, Chen HH, Hsu PY, Chang TY, Ho CL, Tzai TS, Chow NH (2005). Co-expression of RON and MET is a prognostic indicator for patients with transitional-cell carcinoma of the bladder. Br J Cancer.

[45] Maggiora P (2003). The RON and MET oncogenes are co-expressed in human ovarian carcinomas and cooperate in activating invasiveness. Exp Cell Res.

[46] McClaine RJ, Marshal AM, Wagh PK, Waltz SE (2010). Ron receptor tyrosine Kinase activation confers resistance to Tamoxifen in breast cancer cell lines. Neoplasia.

[47] Previdi S, Abbadessa G, Dalo F, France DS, Broggini M (2012). Breast cancer-derived bone metastasis can be effectively reduced through specific c-MET inhibitor tivantinib (ARQ 197) and shRNA c-MET knockdown. Mol Cancer Ther.

[48] Takeuchi H, Bilchik A, Saha S, Turner R, Wiese D, Tanaka M, Kuo C, Wang HJ, Hoon DS (2003). c-MET expression level in primary colon cancer: a predictor of tumor invasion and lymph node metastases. Clin Cancer Res.

[49] Zhao S, Ammanamanchi S, Brattain M, Cao L, Thangasamy A, Wang J, Freeman JW (2008). Smad4-dependent TGF-beta signaling suppresses RON receptor tyrosine kinase-dependent motility and invasion of pancreatic cancer cells. J Biol Chem.

[50] Benvenuti S, Lazzari L, Arnesano A, Li Chiavi G, Gentile A, Comoglio PM (2011). Ron kinase transphosphorylation sustains MET oncogene addiction. Cancer Res.

[51] Gaudino G, Follenzi A, Naldini L, Collesi C, Santoro M, Gallo KA, Godowski PJ, Comoglio PM (1994). RON is a heterodimeric tyrosine kinase receptor activated by the HGF homologue MSP. EMBO J.

[52] Follenzi A, Bakovic S, Gual P, Stella MC, Longati P, Comoglio PM (2000). Cross-talk between the proto-oncogenes met and Ron. Oncogene.

[53] Heldin CH (1995). Dimerization of cell surface receptors in signal transduction. Cell.

[54] Peace BE, Hughes MJ, Degen SJ, Waltz SE (2001). Point mutations and overexpression of Ron induce transformation, tumor formation, and metastasis. Oncogene.

[55] Pinkas-Kramarski R, Shelly M, Glathe S, Ratzkin BJ, Yarden Y (1996). Neu differentiation factor/neuregulin isoforms activate distinct receptor combinations. J Biol Chem.

[56] Zhao S, Cao L, Freeman JW (2013). Knockdown of RON receptor kinase delays but does not prevent tumor progression while enhancing HGF/MET signaling in pancreatic cancer cell lines. Oncogene.

[57] Wang MH, Wang D, Chen YQ (2003). Oncogenic and invasive potentials of human macrophage-stimulating protein receptor, the RON receptor tyrosine kinase. Carcinogenesis.

[58] Chaudhuri A, Xie MH, Yang B, Mahapatra K, Liu J, Marsters S, Bodepudi S, Ashkenazi A (2011). Distinct involvement of the Gab1 and Grb2 adaptor proteins in signal transduction by the related receptor tyrosine kinases RON and MET. J Biol Chem.

[59] Logan-Collins J, Thomas RM, Yu P, Jaquish D, Mose E, French R, Stuart W, McClaine R, Aronow B, Hoffman RM (2010). Silencing of RON receptor signaling promotes apoptosis and gemcitabine sensitivity in pancreatic cancers. Cancer Res.

[60] Velpula KK, Dasari VR, Asuthkar S, Gorantla B, Tsung AJ (2012). EGFR and c-Met cross talk in Glioblastoma and its regulation by human cord blood stem cells. Transl Oncol.

[61] McDermott U, Pusapati RV, Christensen JG, Gray NS, Settleman J (2010). Acquired resistance of non-small cell lung cancer cells to MET kinase inhibition is mediated by a switch to epidermal growth factor receptor dependency. Cancer Res.

[62] Agarwal S, Zerillo C, Kolmakova J, Christensen JG, Harris LN, Rimm DL, Digiovanna MP, Stern DF (2009). Association of constitutively activated hepatocyte growth factor receptor (met) with resistance to a dual EGFR/Her2 inhibitor in non-small-cell lung cancer cells. Br J Cancer.

[63] Breindel JL, Haskins JW, Cowell EP, Zhao M, Nguyen DX, Stern DF (2013). EGF receptor activates MET through MAPK to enhance non-small cell lung carcinoma invasion and brain metastasis. Cancer Res.

[64] Mueller KL, Hunter LA, Ethier SP, Boerner JL (2008). Met and c-Src cooperate to compensate for loss of epidermal growth factor receptor kinase activity in breast cancer cells. Cancer Res.

[65] Ramalingam SS, Owonikoko TK, Khuri FR (2011). Lung cancer: new biological insights and recent therapeutic advances. CA Cancer J Clin.

[66] Wang W, Li Q, Takeuchi S, Yamada T, Koizumi H, Nakamura T, Matsumoto K, Mukaida N, Nishioka Y, Sone S (2012). Met kinase inhibitor E7050 reverses three different mechanisms of hepatocyte growth factor-induced tyrosine kinase inhibitor resistance in EGFR mutant lung cancer. Clin Cancer Res.

[67] Engelman JA, Zejnullahu K, Mitsudomi T, Song Y, Hyland C, Park JO, Lindeman N, Gale CM, Zhao X, Christensen J (2007). MET amplification leads to gefitinib resistance in lung cancer by activating ERBB3 signaling. Science.

[68] Benedettini E, Sholl LM, Peyton M, Reilly J, Ware C, Davis L, Vena N, Bailey D, Yeap BY, Fiorentino M (2010). Met activation in non-small cell lung cancer is associated with de novo resistance to EGFR inhibitors and the development of brain metastasis. Am J Pathol.

[69] Sequist LV, Martins RG, Spigel D, Grunberg SM, Spira A, Janne PA, Joshi VA, McCollum D, Evans TL, Muzikansky A (2008). First-line gefitinib in patients with advanced non-small-cell lung cancer harboring somatic EGFR mutations. J Clin Oncol.

[70] Turke AB, Zejnullahu K, Wu YL, Song Y, Dias-Santagata D, Lifshits E, Toschi L, Rogers A, Mok T, Sequist L (2010). Preexistence and clonal selection of MET amplification in EGFR mutant NSCLC. Cancer Cell.

[71] Raghav KP, Gonzalez-Angulo AM, Blumenschein GR (2012). Role of HGF/MET axis in resistance of lung cancer to contemporary management. Transl Lung Cancer Res.

[72] Kaposi-Novak P, Lee JS, Gomez-Quiroz L, Coulouarn C, Factor VM, Thorgeirsson SS (2006). Met-regulated expression signature defines a subset of human hepatocellular carcinomas with poor prognosis and aggressive phenotype. J Clin Invest.

[73] Ke AW, Shi GM, Zhou J, Wu FZ, Ding ZB, Hu MY, Xu Y, Song ZJ, Wang ZJ, Wu JC (2009). Role of overexpression of CD151 and/or c-Met in predicting prognosis of hepatocellular carcinoma. Hepatology.

[74] Wang ZL, Liang P, Dong BW, Yu XL, Yu DJ (2008). Prognostic factors and recurrence of small hepatocellular carcinoma after hepatic resection or microwave ablation: a retrospective study. J Gastrointest Surg.

[75] Ueki T, Fujimoto J, Suzuki T, Yamamoto H, Okamoto E (1997). Expression of hepatocyte growth factor and its receptor c-met proto-oncogene in hepatocellular carcinoma. Hepatology.

[76] Viticchie G, PAJ M (2015). c-Met and other cell surface molecules: interaction, activation and functional consequences. Biomedicine.

[77] You H, Ding W, Dang H, Jiang Y, Rountree CB (2011). c-Met represents a potential therapeutic target for personalized treatment in hepatocellular carcinoma. Hepatology.

[78] Yarden Y (2001). The EGFR family and its ligands in human cancer. Signalling mechanisms and therapeutic opportunities. Eur J Cancer.

[79] Merchant M, Ma X, Maun HR, Zheng Z, Peng J, Romero M, Huang A, Yang NY, Nishimura M, Greve J (2013). Monovalent antibody design and mechanism of action of onartuzumab, a MET antagonist with anti-tumor activity as a therapeutic agent. Proc Natl Acad Sci U S A.

[80] Gelsomino F, Facchinetti F, Haspinger ER, Garassino MC, Trusolino L, De Braud F, Tiseo M (2014). Targeting the MET gene for the treatment of non-small-cell lung cancer. Crit Rev Oncol Hematol.

[81] Camidge DR, Ou SHI, Shapiro G, Otterson GA, Villaruz LC, Villalona-Calero MA. Efficacy and safety of crizotinib in patients with advanced c-MET-amplified non-small cell lung cancer (NSCLC). 2014.

[82] Shea M, Costa DB, Rangachari D (2016). Management of advanced non-small cell lung cancers with known mutations or rearrangements: latest evidence and treatment approaches. Ther Adv Respir Dis.

[83] Facchinetti F, Rossi G, Bria E, Soria JC, Besse B, Minari R, Friboulet L, Tiseo M (2017). Oncogene addiction in non-small cell lung cancer: focus on ROS1 inhibition. Cancer Treat Rev.

[84] Huang XX, Xie FF, Hou LJ, Chen XX, Ou RY, Yu JT, Qiu JG, Zhang WJ, Jiang QW, Yang Y (2017). Crizotinib synergizes with cisplatin in preclinical models of ovarian cancer. Am J Transl Res.

[85] Stanley A, Ashrafi GH, Seddon AM, Modjtahedi H (2017). Synergistic effects of various her inhibitors in combination with IGF-1R, c-Met and Src targeting agents in breast cancer cell lines. Sci Rep.

[86] Vergote IB, Smith DC, Berger R, Kurzrock R, Vogelzang NJ, Sella A, Wheler J, Lee Y, Foster PG, Weitzman R, Buckanovich RJ (2017). A phase 2 randomised discontinuation trial of cabozantinib in patients with ovarian carcinoma. Eur J Cancer.

[87] Escudier B, Lougheed JC, Albiges L (2016). Cabozantinib for the treatment of renal cell carcinoma. Expert Opin Pharmacother.

[88] Bentzien F, Zuzow M, Heald N, Gibson A, Shi Y, Goon L, Yu P, Engst S, Zhang W, Huang D (2013). In vitro and in vivo activity of cabozantinib (XL184), an inhibitor of RET, MET, and VEGFR2, in a model of medullary thyroid cancer. Thyroid.

[89] Choueiri TK, Escudier B, Powles T, Tannir NM, Mainwaring PN, Rini BI, Hammers HJ, Donskov F, Roth BJ, Peltola K (2016). Cabozantinib versus everolimus in advanced renal cell carcinoma (METEOR): final results from a randomised, open-label, phase 3 trial. The Lancet Oncology.

[90] Shintani T, Kusuhara Y, Daizumoto K, Dondoo TO, Yamamoto H, Mori H, Fukawa T, Nakatsuji H, Fukumori T, Takahashi M, Kanayama H (2017). The involvement of Hepatocyte growth factor-MET-matrix metalloproteinase 1 Signaling in bladder cancer invasiveness and proliferation. Effect of the MET inhibitor, Cabozantinib (XL184), on bladder cancer cells. Urology.

[91] Shah MA, Wainberg ZA, Catenacci DV, Hochster HS, Ford J, Kunz P, Lee FC, Kallender H, Cecchi F, Rabe DC (2013). Phase II study evaluating 2 dosing schedules of oral foretinib (GSK1363089), cMET/VEGFR2 inhibitor, in patients with metastatic gastric cancer. PLoS One.

[92] Choueiri TK, Vaishampayan U, Rosenberg JE, Logan TF, Harzstark AL, Bukowski RM, Rini BI, Srinivas S, Stein MN, Adams LM (2013). Phase II and biomarker study of the dual MET/VEGFR2 inhibitor foretinib in patients with papillary renal cell carcinoma. J Clin Oncol.

[93] Chia SK, Ellard SL, Mates M, Welch S, Mihalcioiu C, Miller WH, Gelmon K, Lohrisch C, Kumar V, Taylor S (2017). A phase-I study of lapatinib in combination with foretinib, a c-MET, AXL and vascular endothelial growth factor receptor inhibitor, in human epidermal growth factor receptor 2 (HER-2)-positive metastatic breast cancer. Breast Cancer Res.

[94] Cheng H, Chua V, Liao C, Purwin TJ, Terai M, Kageyama K, Davies MA, Sato T, Aplin AE (2017). Co-targeting HGF/cMET Signaling with MEK inhibitors in metastatic Uveal melanoma. Mol Cancer Ther.

[95] Barat S, Bozko P, Chen X, Scholta T, Hanert F, Gotze J, Malek NP, Wilkens L, Plentz RR (2016). Targeting c-MET by LY2801653 for treatment of cholangiocarcinoma. Mol Carcinog.

[96] Wang J, Cheng JX (2017). c-Met inhibition enhances chemosensitivity of human ovarian cancer cells. Clin Exp Pharmacol Physiol.

[97] Liu X, Wang Q, Yang G, Marando C, Koblish HK, Hall LM, Fridman JS, Behshad E, Wynn R, Li Y (2011). A novel kinase inhibitor, INCB28060, blocks c-MET-dependent signaling, neoplastic activities, and cross-talk with EGFR and HER-3. Clin Cancer Res.

[98] Okusaka T, Aramaki T, Inaba Y, Nakamura S, Morimoto M, Moriguchi M, Sato T, Ikawa Y, Ikeda M, Furuse J (2015). Phase I study of tivantinib in Japanese patients with advanced hepatocellular carcinoma: distinctive pharmacokinetic profiles from other solid tumors. Cancer Sci.

[99] Xiang Q, Zhen Z, Deng DY, Wang J, Chen Y, Li J, Zhang Y, Wang F, Chen N, Chen H, Chen Y (2015). Tivantinib induces G2/M arrest and apoptosis by disrupting tubulin polymerization in hepatocellular carcinoma. J Exp Clin Cancer Res.

[100] Best J, Schotten C, Lohmann G, Gerken G, Dechene A (2017). Tivantinib for the treatment of hepatocellular carcinoma. Expert Opin Pharmacother.

[101] Sequist LV, von Pawel J, Garmey EG, Akerley WL, Brugger W, Ferrari D, Chen Y, Costa DB, Gerber DE, Orlov S (2011). Randomized phase II study of erlotinib plus tivantinib versus erlotinib plus placebo in previously treated non-small-cell lung cancer. J Clin Oncol.

[102] Yoshioka H, Azuma K, Yamamoto N, Takahashi T, Nishio M, Katakami N, Ahn MJ, Hirashima T, Maemondo M, Kim SW (2015). A randomized, double-blind, placebo-controlled, phase III trial of erlotinib with or without a c-Met inhibitor tivantinib (ARQ 197) in Asian patients with previously treated stage IIIB/IV nonsquamous nonsmall-cell lung cancer harboring wild-type epidermal growth factor receptor (ATTENTION study). Ann Oncol.

[103] Scagliotti G, von Pawel J, Novello S, Ramlau R, Favaretto A, Barlesi F, Akerley W, Orlov S, Santoro A, Spigel D (2015). Phase III multinational, randomized, double-blind, placebo-controlled study of Tivantinib (ARQ 197) plus Erlotinib versus Erlotinib alone in previously treated patients with locally advanced or metastatic Nonsquamous non-small-cell lung cancer. J Clin Oncol.

[104] Puzanov I, Sosman J, Santoro A, Saif MW, Goff L, Dy GK, Zucali P, Means-Powell JA, Ma WW, Simonelli M (2015). Phase 1 trial of tivantinib in combination with sorafenib in adult patients with advanced solid tumors. Investig New Drugs.

[105] Klempner SJ, Borghei A, Hakimian B, Ali SM, Ou SI (2017). Intracranial activity of Cabozantinib in MET exon 14-positive NSCLC with brain metastases. J Thorac Oncol.

[106] Pedersen L, Christensen JF, Hojman P (2015). Effects of exercise on tumor physiology and metabolism. Cancer J.

[107] Corbet C, Feron O (2017). Tumour acidosis: from the passenger to the driver's seat. Nat Rev Cancer.

[108] Fan C, Tang Y, Wang J, Xiong F, Guo C, Wang Y, Zhang S, Gong Z, Wei F, Yang L (2017). Role of long non-coding RNAs in glucose metabolism in cancer. Mol Cancer.

[109] Wang Y, Mo Y, Gong Z, Yang X, Yang M, Zhang S, Xiong F, Xiang B, Zhou M, Liao Q (2017). Circular RNAs in human cancer. Mol Cancer.

[110] Song Y, Li X, Zeng Z, Li Q, Gong Z, Liao Q, Li X, Chen P, Xiang B, Zhang W (2016). Epstein-Barr virus encoded miR-BART11 promotes inflammation-induced carcinogenesis by targeting FOXP1. Oncotarget.

